# Assessing medical students’ perception of cross-cultural competence at a private University in Karachi

**DOI:** 10.1186/s12909-022-03588-0

**Published:** 2022-07-09

**Authors:** Fatima Syed Amanullah, Adil Al-Karim Manji, Bilal Ahmed Usmani, Muhammad Muntazir Mehdi Khan, Hadia Sohail, Muhammad Haris Zahid, Meryum Ishrat Baig, Inara Merani, Shehryar Ali Larik, Shahmeer Raza Khan, Syeda Ramlah Tul Sania

**Affiliations:** 1 The Aga Khan University Hospital, Karachi, Pakistan; 2 NED University of Engineering & Technology, Karachi, Pakistan

**Keywords:** Cross-cultural competence, Diversity, Medical education, Students, Knowledge, Comfort, Skills

## Abstract

**Background:**

Cross-cultural competence is widely regarded to play an important role in being able to deliver appropriate and effective health care to patients with different backgrounds, race, gender orientation and cultural beliefs. This study aims to assess how medical students feel about their comfort, knowledge, and skill level in handling a diverse patient population using a validated questionnaire.

**Methods:**

This study was carried out over a period of three weeks from July 5th to July 26^th^ of 2021, in the Aga Khan University Hospital, Karachi, Pakistan. All medical students who fulfilled the eligibility criteria and gave informed consent were included in the study. A modified version of the Harvard cross-cultural care survey was used to assess the medical students’ comfort, knowledge and skill level in a variety of circumstances related to patients with different backgrounds and cultures. Descriptive statistical analysis of the questionnaire items was carried out. We reported frequencies and percentages for gender and year of study. For the questionnaire items, we reported mean, assuming that our Likert scale had equivariant intervals. Furthermore, multivariate analysis between demographics and themes was carried out. A p-value of < 0.05 was taken as statistically significant.

**Results:**

It was found that students of year 5 considered themselves more knowledgeable, comfortable and skilled in dealing with patients of different backgrounds, religions and beliefs compared to students of year 1 and had a higher average score in all of these categories which was statistically significant. Additionally, students who believed it is extremely important to practice medicine with a diverse patient population also had the highest averages in perceived knowledge, comfort and skills in dealing with patients of different sociocultural backgrounds compared to students who believed it wasn’t important at all.

**Conclusion:**

This is a first of its kind study in a private medical university in Pakistan and highlights the students’ self-assessment of their competence when caring for patients from different backgrounds. This study can be used as a reference study in the region to carry out further studies and to assess and improve the gaps in medical training being provided.

**Supplementary Information:**

The online version contains supplementary material available at 10.1186/s12909-022-03588-0.

## Background

With the improvement of the healthcare system, research has found that rather than having a generic approach to each patient, patient-centered care is an effective method in order to ensure patient satisfaction, compliance, and better healthcare outcomes. In order to provide patient centered care, cross-cultural competence is an essential skill in health care professionals, and therefore must be taught alongside other basic clinical skills within the medical curriculum [1].

Research has shown that cross-cultural competence is widely regarded to play an important role in medical students being able to deliver appropriate care to patients with different backgrounds, race, gender orientation and cultural beliefs [1]. Karachi is one of the largest cities in the world, and contains a multitude of ethnicities, religious sects, and people from diverse socioeconomic backgrounds, with varying levels of education [2]. From its very existence, the city of Karachi has been considered home to a diverse range of cultural, religious and ethnic groups. With the rise of globalization, Karachi has witnessed the immigration of many Middle Eastern, Afghan and Central Asian migrants, and is home to a number of different religious communities including Muslims, Hindus, Christians and Sikhs, earning its title as the “Melting Pot” of Pakistan [3]. The city’s population is approximately 23 million with 90% of inhabitants being immigrants [4]. A national survey conducted in 2017 showed that about 42% of the population spoke primarily Urdu, 15% spoke Pashto, 11% spoke Punjabi and 11% spoke Sindhi. The remaining fourteen languages spoken are a mix of local and regional dialects each having their own unique form, content and use [5]. A large majority of the city’s population consists of migrants, consisting of Pashtuns, Afghan refugees, and many other groups [6]. Much of the population also resides in *katchi abadis,* or slums [7]. The population groups most affected by this are those with recent migrations or cultural heterogeneity which results in the people having a wide variety of beliefs and values. This variation can be challenging for a medical student to deal with and in ensuring patient satisfaction [8]. In order to effectively treat this diverse patient population, cross-cultural competence is a necessary skill for all medical students.

Successful implementation of cross-cultural competence has been shown to be essential in developing healthcare professionals who are prepared to deal with a variety of clinical scenarios with patients of different identities and beliefs [2]. A study done at Harvard Medical School showed that students in their final year of medical college felt that they were not adequately prepared to deal with important aspects of cross-cultural care [1]. Similarly, in 2003 another study was performed in the United States with the aim of assessing physician readiness in providing cross-cultural care. Through a survey distributed to many US academic health centers, it was found that 25% of senior residents felt unprepared to provide care to new immigrants and patients with odd health beliefs while 20% were unable to provide adequate care to patients having religious beliefs that affect treatment. Apart from these issues, language barriers have also been a hindrance for physicians in training while providing patient centered care [9].

The accreditation council for graduate medical education requires cultural competency in two of its components—professionalism and interpersonal/communication skills. They require residents to show respect, compassion and understanding towards all patients without any discrimination. However, it is difficult to assess cultural competency due to its subjectivity. One method to assess cross-cultural competence is through a cross-cultural care survey. It has been noted that self-assessments for cultural competency can increase knowledge, positive attitudes and awareness after training, whereas other objective assessments show variety in effectiveness [10].

The initial need to address cross-cultural competence originated in Western countries’ medical education. Consequently, much of the research to date has focused mainly on Western contexts, and is primarily reflective of local concerns [11]. There are fewer relevant studies in areas such as certain regions of Asia. However, the need for healthcare providers to recognize, respect and treat a diverse patient population remains [8]. There has not been any study conducted to evaluate cross-cultural competence in Pakistani medical students and health care providers. In a city like Karachi, this skill is especially important to ensure that patients with diverse backgrounds feel comfortable and that students can be a part of providing quality medical care. The information gained from studies like these can be integral in determining whether medical students in Karachi are ready to deal with patients of diverse ethnicities, cultures, and backgrounds and if there are certain aspects of cross-cultural care in which they do not feel confident. It will ensure that the medical school produces well rounded doctors who can deal with multiple scenarios involving patients of different beliefs and values.

These themes were identified by a questionnaire in which medical students assessed their preparedness to treat patients from different backgrounds and beliefs. Within the concept of preparedness, we focused on three main aspects of competence – knowledge, comfort level, and skill set. The questionnaire was based completely on self-reflection as the intention of the survey was to determine how students view their current state of competence in a cross-cultural context. It also served the purpose of determining how much importance students give to cultural diversity in a medical setting as well as the need for recognizing barriers that may reduce the ability to give quality medical care.

## Methodology

A cross-sectional study was carried out to determine medical students’ perception of knowledge, comfort level and skill in handling a diverse patient population. We selected the Aga Khan University Hospital Karachi as it provides education to students from all over Pakistan as well as from countries outside Pakistan. Therefore, we believe that it would represent a balanced sample for the population due to diversity of the student body. All medical students at the Aga Khan University Hospital, Karachi, Pakistan, who fulfilled the eligibility criteria and gave informed consent were included in the study. The study was conducted in a period of three weeks from July 5th to July 26th, 2021 after getting approval from the Ethics and Review Committee of the Aga Khan University. The sample size was calculated to be 218 students for the study. It was calculated using OpenEpi software. The population size taken was 500 (number of total medical students in Aga Khan University Hospital Medical College). A confidence interval of 95% was used. As no study related to ours had been done on a national level, the anticipated percentage frequency of students’ participation was 50%. The students of all five medical years, aged 18 years and above were included in the study.

An online questionnaire was created using Google forms. The questionnaire used in this study closely follows relevant sections of the Harvard cross-cultural care survey (1). The questionnaire was modified to make each question relevant to our setting. The language used for the questionnaire was English because students at the medical college are quite proficient in English language. A consent form was provided before starting the questionnaire. The questionnaire was sent out via email from the medical college database. We further circulated the questionnaire on Social Media platforms. Once data was collected via the Google Forms, preliminary analyses were performed. Data was then stored in SPSS for further evaluation. Forms with missing data were filtered out from the analysis process to maintain the quality of data. The questionnaire contained basic demographic questions about age, gender, year of study, languages spoken, and town of origin. A pilot study was conducted first using a sample size of 40 in in order to establish the validity of our study.

To maintain the confidentiality of the respondents, no names were requested while collecting the data. However, to ensure that the questionnaire was not spammed, and there was one response per an email address, the ‘limit to 1 response’ feature in Google Forms was used, which prevented duplicate entries without disclosing the email addresses of participants. In order to ensure that the data was not misused, the responses were accessible only to the principal author, who disseminated data to the analysis team. Students’ confidentiality was maintained throughout the study. The data was analyzed using the IBM SPSS software. For qualitative variables such as gender, year of study, origin and language spoken, frequencies and percentages were reported. For the questionnaire items, we reported means and standard deviations. Furthermore, multivariate analysis between demographics and themes was carried out. Independent t tests were used to compare the means between different groups.

### Eligibility criteria

Students of all five medical years from Aga Khan University Hospital Medical College were included in the study. Only participants of age 18 and above were included.

### Exclusion criteria

Students of year 4 who are Co-Investigators in this study are excluded. Participants of age 17 and below were excluded.

### Operational definitions

Knowledge: Understanding, information, awareness and/or familiarity about something, such as a certain subject or situation, often acquired through education or experience.

‘Cross-Cultural Competence (CCC)’ – the ability of individuals to communicate effectively with those from another culture, language, religion, socioeconomic status, or education level [1, 8].

‘Culture’ is defined as the languages, customs, beliefs, rules, arts, knowledge, collective identities, and memories developed by members of all social groups that make their social environments meaningful [1, 8].

‘Cultural sensitivity’ is also related to the concept of cross-cultural competence in regards to the attitude and willingness of medical professionals in caring for a diverse population [12].

In our study, we use cross-cultural competence as an umbrella term for intercultural competence and transcultural competence.

Comfort is defined as a pleasant feeling associated with confidence, willingness and a relaxed mindset. Comfort level is assessed using the Likert scale.

## Results

**Fig. 1:**
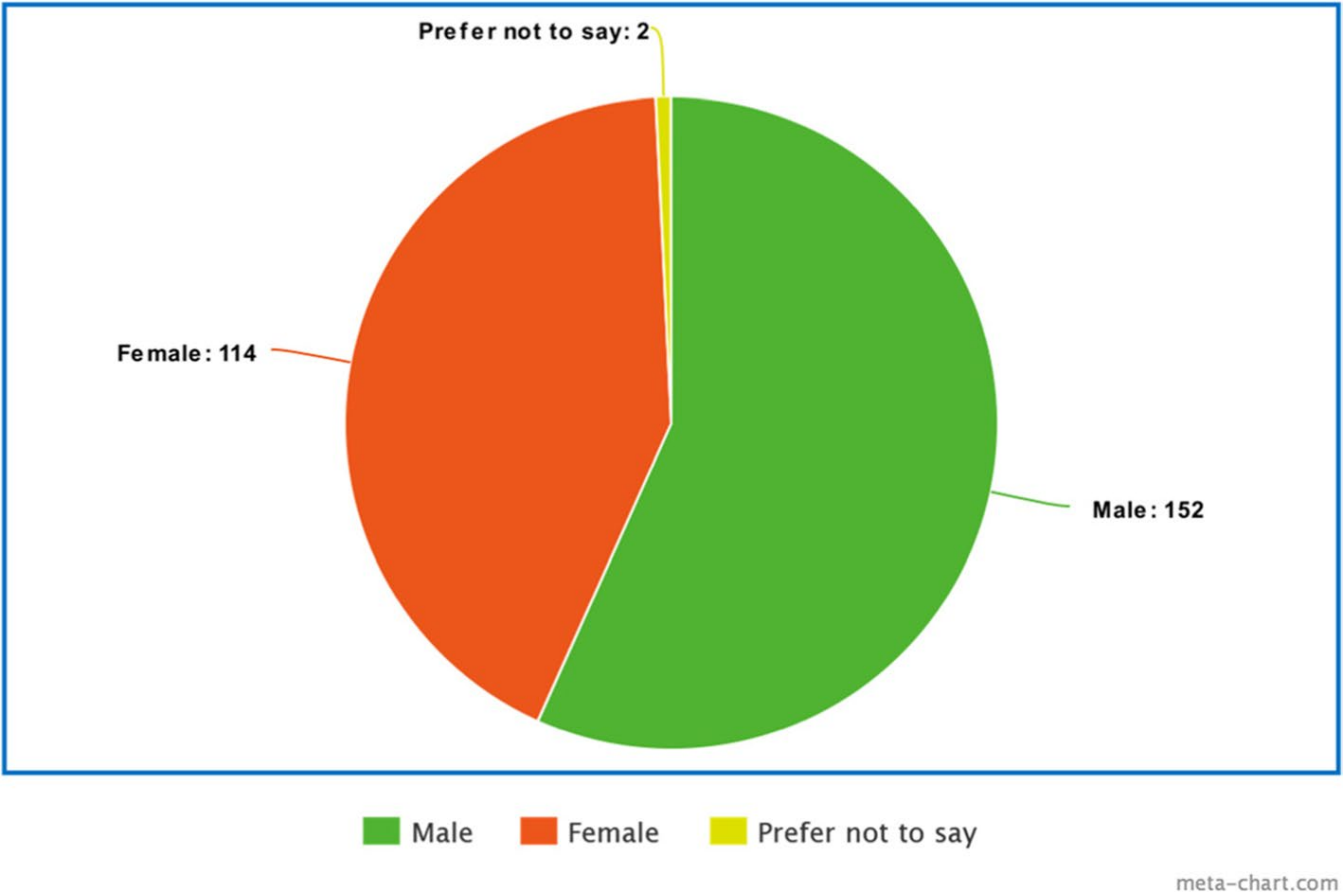
Gender distribution of participants

**Fig. 2:**
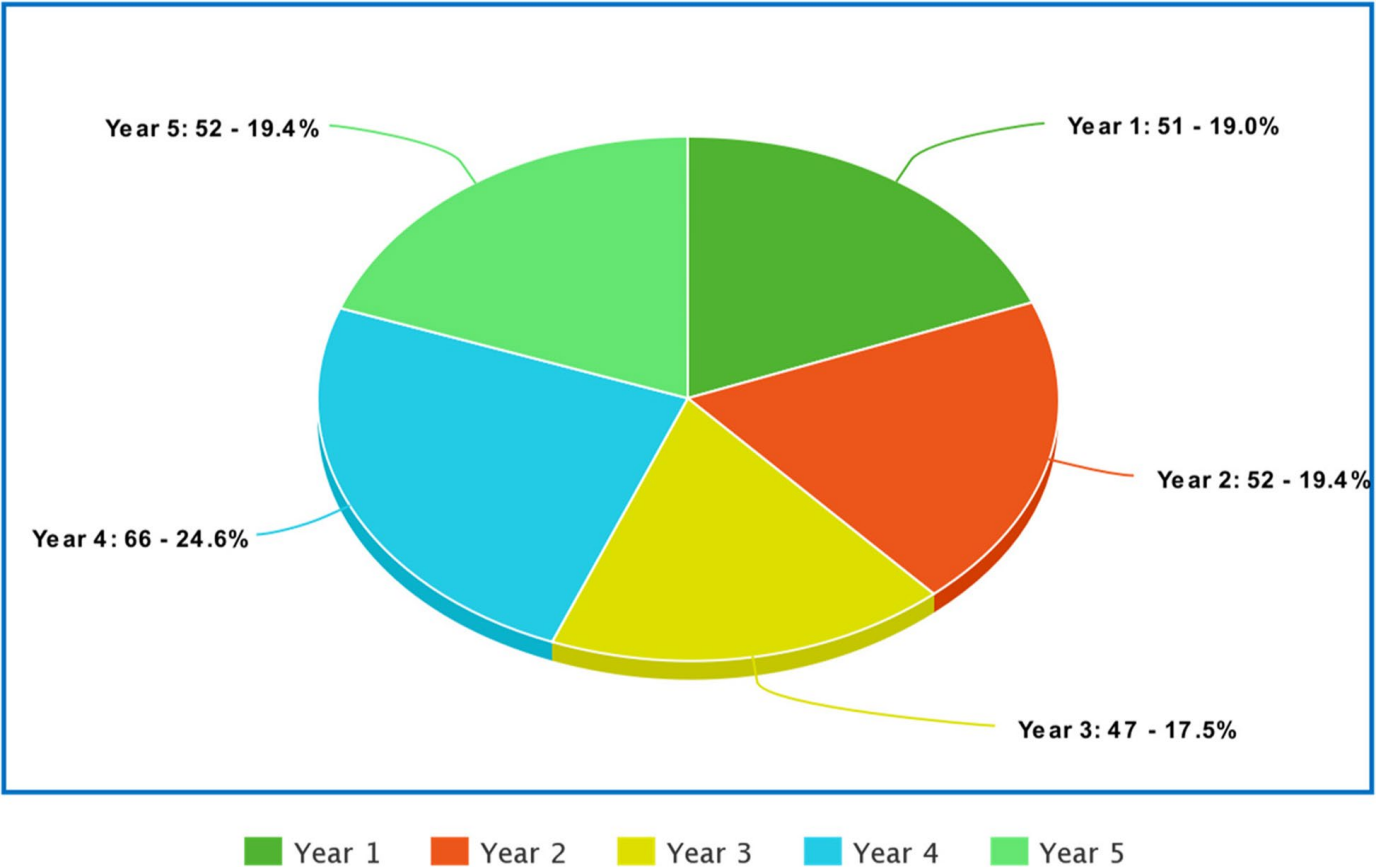
Year of study of participants

Figures 1 and 2 are visual representations of the demographics of the students who participated in the study. Figure 1 shows that of the 268 participants, approximately 57% were male while 43% were female. Figure 2 shows that almost an equal number of students from each year participated in the study ranging from 47 to 66 students.

According to Fig. 2, our study sample had an almost equal percentage of students from each year of medical college. Table 1 demonstrates that perceived *knowledge, comfort* and *skills* related to dealing with patients from different sociocultural backgrounds, different religions, alternate medicine and different languages mean scores increased across the year of study. Each theme was assessed using a Likert scale where the responses were graded from 1 to 5. Having little to no knowledge, comfort or skill was graded as 1. Whereas if students felt that they had superior knowledge or they felt extremely skilled or comfortable in these components of competence, their responses would be graded as 5. In our study, the means for perceived skills regarding patients from different sociocultural backgrounds, different religions, alternate medicine and different languages, were 2.28, 2.29, 2.10 and 1.94 respectively for year 1. However, the means were 3.53, 3.72, 3.16, and 2.90 respectively for year 5. The means were approximately 3 (moderate) for the majority of themes across all five years. For the preclinical years (year 1 and 2), the means were less than 3.4 for most themes, but for clinical years (year 3, 4 and 5), the means were above 3.5 for the majority of the themes.

**Table 1:** Comparing year of study with perceived abilities

	Year of Study									
	Year 1		Year 2		Year 3		Year 4		Year 5	
	Mean	Standard Deviation	Mean	Standard Deviation	Mean	Standard Deviation	Mean	Standard Deviation	Mean	Standard Deviation
Knowledge: SC	2.93	.72	3.33	.52	3.49	.53	3.77	.50	3.82	.57
Comfort: SC	3.29	.73	3.13	.79	3.35	.62	3.66	.75	3.65	.75
Skill: SC	2.28	.94	2.66	.78	3.13	.62	3.30	.62	3.53	.73
Knowledge: religion	2.99	.78	3.25	.67	3.52	.67	3.91	.67	3.91	.56
Comfort: religion	3.35	.97	3.20	.75	3.30	.60	3.79	.81	3.75	.80
Skill: religion	2.29	.90	2.79	.85	3.25	.59	3.58	.76	3.72	.76
Knowledge: alternative medicine	2.71	1.04	3.00	.91	3.38	.84	3.30	.77	3.53	.79
Comfort: alternative medicine	3.19	.92	2.84	.88	3.10	.80	3.14	.81	3.30	.72
Skill: alternative medicine	2.10	1.00	2.51	.89	2.86	.77	2.92	.71	3.16	.72
Comfort: language	2.61	.77	2.88	.57	2.85	.81	2.81	.67	2.97	.93
Skill: language	1.94	.94	2.47	.72	2.59	.80	2.75	.69	2.90	.94

The highest means were noted in the categories assessing medical students’ *knowledge, skills* and *comfort* when dealing with patients from a different religious background. This result was consistent in medical students from all 5 years.

**Fig. 3:**
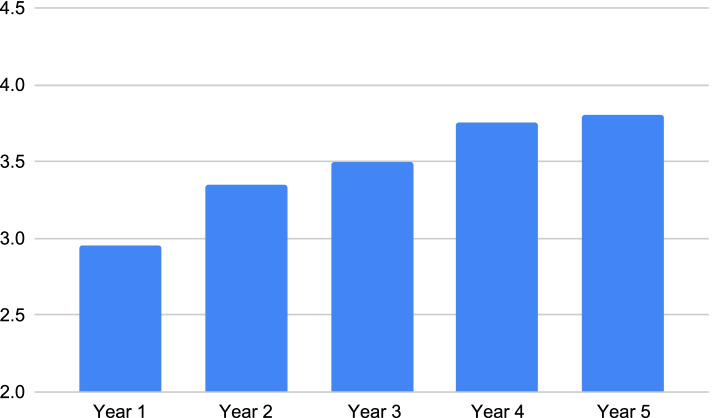
Students’ perceived knowledge regarding patients’ sociocultural background according to year of study

**Fig. 4:**
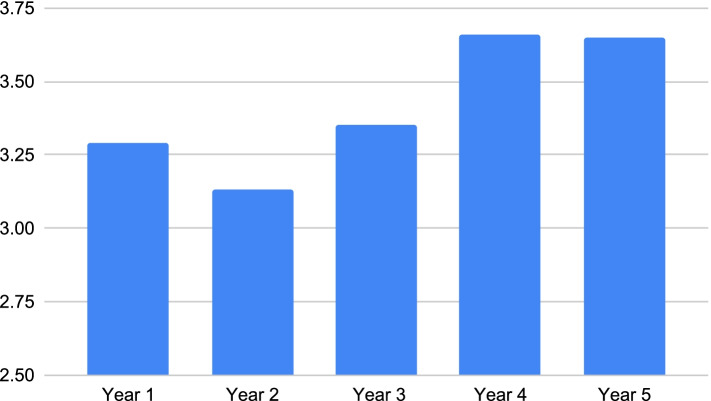
Students’ perceived skill regarding patients’ sociocultural background according to year of study

**Fig. 5:**
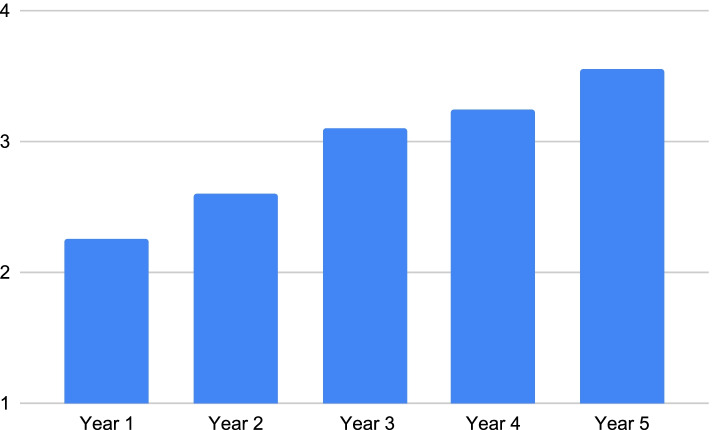
Students’ perceived comfort regarding patients’ sociocultural background according to year of study

Figures 3 and 4 are pictorial representations of the average perceived knowledge, comfort and skill levels that students from each year had in regards to providing care to patients with different sociocultural backgrounds. In Figures. 3 and 4, a steady upward trend in perceived skill can be noted with each progressing year. However, Fig. 5 shows a drop in perceived comfort of second year students in treating patients with different sociocultural backgrounds.

Table 2 shows that among all the participants, the majority (91.8%) were from Pakistan and the remainder (8.2%) participants were international students. We analyzed these two groups based on their knowledge, comfort and skill in dealing with themes mentioned in the study. According to Table 3, the mean value on the Likert scale for all the components were similar in both the groups. The mean values were higher for the group whose place of origin was outside Pakistan in all the components except comfort and knowledge while dealing with people from different religions.

**Table 2:** Participant origin

Origin			
	Frequency	Percent	Cumulative Percent
Other than Pakistan	22	8.2	8.2
Pakistan	246	91.8	100.0
Total	268	100.0

**Table 3:** Comparing perceived abilities based on primary language spoken and origin

	Language				Origin			
	Other		Urdu		Other		Pakistan	
	Mean	Standard Deviation	Mean	Standard Deviation	Mean	Standard Deviation	Mean	Standard Deviation
Knowledge: SC	3.51	.69	3.47	.63	3.52	.78	3.48	.64
Comfort: SC	3.47	.85	3.40	.68	3.53	.76	3.42	.76
Skill: SC	3.04	.90	2.96	.83	3.30	.71	2.97	.87
Knowledge: religion	3.61	.76	3.49	.76	3.39	.66	3.55	.77
Comfort: religion	3.53	.87	3.47	.80	3.34	.66	3.51	.84
Skill: religion	3.21	.96	3.10	.92	3.39	.65	3.13	.95
Knowledge: alternative medicine	3.19	.88	3.19	.94	3.45	.65	3.16	.93
Comfort: alternative medicine	3.18	.84	3.06	.83	3.23	.84	3.10	.84
Skill: alternative medicine	2.75	.92	2.70	.87	2.77	.72	2.72	.91
Comfort: language	2.76	.77	2.87	.74	2.83	.77	2.82	.76
Skill: language	2.54	.89	2.54	.87	2.87	.88	2.51	.87

We also formed two groups based on the primary language that participants speak. The first group was of participants who speak Urdu (56%). The second group was of participants who speak other languages (44%) as seen in Table 4. We analyzed these two groups based on their knowledge, comfort and skills in dealing with themes mentioned in the study. The mean value on the Likert scale for all the components were similar in both groups. The mean values were slightly higher for the group whose primary language was not Urdu in all the components except for the level of comfort while dealing with patients who speak different languages than them.

**Table 4:** Participant primary language

Language			
	Frequency	Percent	Cumulative Percent
Other than Urdu	118	44.0	44.0
Urdu	150	56.0	100.0
Total	268	100.0	

**Fig. 6:**
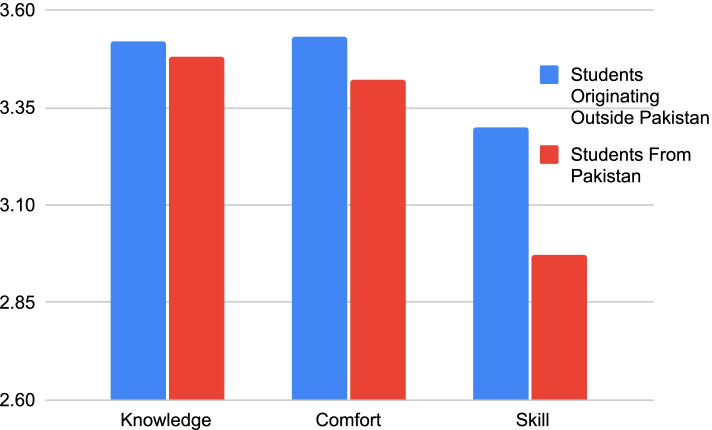
Comparing student origin with perceived knowledge, comfort and skills

Figure 6 shows that students originating from outside Pakistan consistently scored higher in all aspects of their perceived abilities in treating patients with different sociocultural backgrounds.

In Table 5 students who believed it is extremely important to practice medicine with a diverse patient population had reported an average skill score of 3.13 whereas students who did not believe it to be important at all had an average skill score of 2.50. The same trend can be seen in Table 6. Students who strongly agreed that healthcare professionals should have a basic understanding of the various groups that make a society reported an average of 3.54 in their knowledge of patients with different sociocultural backgrounds. Comparatively, those who strongly disagreed on this statement reported an average of 3.00 regarding their knowledge.

**Table 5:** Comparing beliefs about practicing in diverse populations with perceived abilities

	I: Practice with a diverse population									
	Extremely		Mildly		Moderately		Not at all		Very	
	Mean	Standard Deviation	Mean	Standard Deviation	Mean	Standard Deviation	Mean	Standard Deviation	Mean	Standard Deviation
Knowledge: SC	3.66	.66	3.22	.29	3.25	.51	3.67	.58	3.36	.68
Comfort: SC	3.70	.72	2.47	.78	3.06	.68	2.50	.43	3.37	.66
Skill: SC	3.13	.96	2.75	.57	2.90	.58	2.50	.43	2.89	.86

**Table 6:** Comparing beliefs about understanding diversity with perceived abilities

	A: Have an understanding about various groups of society									
	Agree		Disagree		Neutral		Strongly agree		Strongly disagree	
	Mean	Standard Deviation	Mean	Standard Deviation	Mean	Standard Deviation	Mean	Standard Deviation	Mean	Standard Deviation
Knowledge: SC	3.49	.64	3.07	.56	3.22	.61	3.54	.66	3.00	.57
Comfort: SC	3.40	.70	2.69	.56	2.87	.40	3.59	.76	1.88	1.24
Skill: SC	2.97	.85	2.78	.58	2.79	.40	3.06	.92	3.00	1.06
Knowledge: religion	3.52	.79	3.22	.72	3.04	.49	3.63	.75	3.38	.88
Comfort: religion	3.45	.79	3.14	.64	3.01	.62	3.63	.86	2.25	.35
Skill: religion	3.21	.91	2.92	.66	3.01	.39	3.15	1.01	2.38	.18
Knowledge: alternative medicine	3.15	.83	3.22	1.00	3.47	.70	3.20	.97	1.75	1.06
Comfort: alternative medicine	3.18	.74	2.50	.66	2.97	.51	3.14	.91	1.75	1.06
Skill: alternative medicine	2.76	.86	2.78	.67	2.91	.36	2.67	.96	2.00	1.41
Comfort: language	2.83	.72	2.81	.76	2.93	.56	2.81	.81	2.75	.35
Skill: language	2.57	.85	2.78	.57	3.00	.60	2.45	.92	2.25	.35

## Discussion

In this study, we identified the themes that medical students felt least prepared for and consequently most hesitant when encountering such patients. We collected 269 responses from our participants, and excluded 1 response in the data analysis. There was a minimum of 44 responses from each year of study. Certain trends were noted in the average scores of knowledge, comfort and skill regarding the themes that were investigated. It was noted that students of year 5 considered themselves more knowledgeable, comfortable and skilled in dealing with patients of different sociocultural backgrounds, religions, languages and beliefs about alternative medicine compared to students of year 1. This pattern could be due to more clinical exposure over the 5 year program offered at the university. The progression from preclinical (years 1 and 2) to clinical (years 3 to 5) was visible in the difference in means of knowledge, comfort and skill. A similar trend was seen in another study performed in Taiwan in 2020. Their results showed that there was a significant drop in reported preparedness in students in preclinical years due to intense focus on academics as opposed to the other components of patient care [8].

After running the independent sample T test, we observed that the difference in the means of year 1 and 5 with respect to knowledge, comfort and skill regarding sociocultural background language and religion was significant. This further strengthens our previous finding that with progressing years, students tend to become more confident in dealing with these types of patients. Starting from year 3, students transition to their clinical years where they spend majority of their time in clinics at the Aga Khan University Hospital. This hospital provides care to a variety of patients originating from different cities in Pakistan. With a population of 16.5 million, Karachi is one of the world’s most populous cities (9). Due to this diverse patient population, students gain exposure to people from other cultures, religions, languages and beliefs. Therefore, once students have entered their final year at the university, they are more competent in handling these patients.

It is important to note that the difference in responses with respect to comfort regarding patients with certain beliefs revolving around alternative medicine was not statistically significant. There is little improvement over the five years regarding comfort handling patients who prefer alternative medicine and handling patients whose beliefs contradict with current evidence-based medicine. It is important to remember that many people in Pakistan have strong beliefs in spiritual healers, hakeems, homeopathic doctors and more [13]. Hence, it can be difficult to counsel such patients into considering conventional or evidence-based medicine. This may be an area that needs to be emphasized in the training process of medical students.

When assessing the relationship between origin and competence in the four themes of our study, we found that students originating outside Pakistan assessed themselves as slightly more competent in dealing with patients from different sociocultural backgrounds, languages and beliefs. These students often originate from multicultural cities outside Pakistan which may account for the increased sense of competence in these areas. As mentioned earlier, the environment plays a significant role in the development of cross-cultural competence [8]. It was seen that students originating within Pakistan scored slightly higher in knowledge and comfort in dealing with patients from different religions. This could be due to emphasis on religion in Pakistani society resulting in local students being more aware of the sensitivity regarding the subject. Islamic Studies is part of the curriculum in Pakistan’s education system, therefore students raised in Pakistan tend to be more conscious of religious limitations. Despite the slight difference between the responses of both groups, it is not considered to be statistically significant.

Our findings are supported by a study done at the Harvard Medical School where fourth year students’ rating of skillfulness in counseling patients who use alternative medicine was significantly low. In addition, there was a noticeable deficiency in skills regarding the recognition of religious beliefs that may affect clinical care [1].

We then analyzed the relationship between primary language and competency. Our results show that students whose primary language is not Urdu, scored themselves slightly higher in all components of competency when handling patients of different sociocultural backgrounds, religions, and beliefs compared to those who chose Urdu as a first language. However, students whose primary language is not Urdu scored slightly lower in comfort levels while dealing with patients who speak a language different than their own. A possible reason for this could be that students who speak Urdu as their first language may have a better understanding of other local languages spoken in Pakistan such as Punjabi and Sindhi considering they share similarities. Again, despite these slight differences, the results are not considered to be significant.

Students who believed it is extremely important to practice medicine with a diverse patient population also had the highest averages in perceived knowledge, comfort and skills in dealing with patients of different sociocultural backgrounds compared to students who believed it was not important at all. Similar trends were seen in response to questions regarding students’ awareness of their biases and limitations, recognizing factors that predisposes to disease, and being considerate of a patient’s culture. Students who believed that these components of care are less important had reported a lower average in their knowledge, comfort and skills. This strengthens the need for universities to develop such beliefs in their medical students to ensure quality patient care.

A “quick fix” to improve cross-cultural competence of medical students would not work, hence a long-term solution is required. It is deduced through the study that the “dosage,” or the length of exposure and training provided may be an important factor impacting practice and perception of the provider. Preparing medical students to deal with patients of different sociocultural backgrounds, religion, language and beliefs might not prove to be sustainable. Pakistan has a diverse patient population from different provinces, each having different cultures and subcultures. Aga Khan University Hospital (AKUH), being a well-known and respected institution, serves this set of culturally diverse people. It should be noted that patients from neighboring countries such as China, Iran and Afghanistan also receive treatment from AKUH. Hence, it will be difficult to train medical students to increase their cross-cultural competence. Another possible solution could be recruiting more medical students from different cultural backgrounds in Pakistan and from the neighboring countries as well. However, this would be a risky approach as it would be unfair for students who are academically competent but are from countries further away from Pakistan. A third novel approach can be towards patient-centered care. In this patient-partner model, each student can be paired with a chronically diseased patient of a certain sociocultural background, language, religion and beliefs. AKUH already practices simulation based medical communication practice, so we already know that this model is feasible and sustainable. Moreover, the PAIRS program study has shown that students can indeed be paired with chronically ill patients for better learning and improvement of professional development in sociocultural background, language, religion and beliefs. This would increase medical student knowledge and positive attitudes towards patients [14, 15]. The model could be adapted to improve medical students’ cross-cultural competence through the establishment of a “cultural-partner” who could be from various ethnic backgrounds. Perhaps the most feasible method to increase cross-cultural competence in students involves introducing regular field visits where students can participate in clinics catering to different sub-populations within the city. This would ensure that students still interact with patients who are unable to visit the main hospital, allowing for greater exposure to the various communities in Karachi.

The findings from this study lay the groundwork for future exploration on the topic of cross-cultural competence in students. With a baseline assessment of cross-cultural competence complete, this study invites further research to help identify gaps in student knowledge, comfort and skill as well as possible interventions. Review of these findings can help educational departments decide any changes that need to be made in the curriculum to strengthen the cross-cultural competence of students. The study also highlights the need for other medical schools to conduct similar assessments on their students to identify areas in training that require improvement.

### Strengths

Our study is a first of its kind in Pakistan. No other study has targeted medical students’ cross-cultural competence in Pakistan yet. It is an important topic to address to help increase the level of healthcare and comfort provided to patients. This study can be used as a model to further develop different questionnaires and understand the level of cross-cultural competence amongst medical students in Pakistan.

There were four different themes that were assessed in this study. This gave us an opportunity to compare different themes to find an association. To get an in-depth response from the participants, the questionnaire used in the study was designed in a way to address all the aspects of a certain theme that was being discussed. Additionally, some questions included examples making it more clear for participants to understand the topic we were assessing.

As we distributed the questionnaire amongst each year of study at the same time, we were able to get a roughly equal number of participants from each year of study, allowing uniform representation. This helped us to compare results from different years of study and make an association between cross-cultural competence and increasing years of study.

The study setting is a well-established University Hospital with a relatively high patient influx from different sociocultural backgrounds. The interaction of students with a diverse group of patients helps in assessing their abilities and comfort level while treating them in a better way.

### Limitations

Our study is limited to the Aga Khan University Hospital and its medical students only, when ideally it should be distributed through other medical schools across Pakistan. Due to this limitation, our results are not an accurate representation of medical students nation-wide.

The study setting was limited to one institute and hence the variety of students was also limited. There was a relatively low representation of students having origin outside of Pakistan. We would have ideally preferred an equal number of students from both the groups to draw definitive conclusions.

Our study should have a five year time span instead of a cross-sectional study at one point in time. The same students should be monitored throughout their five year experience. This approach would have reduced bias in assessing students’ perception of cross-cultural competence.

Due to the current COVID-19 pandemic climate, results may be skewed or biased towards decreased competency simply because many students were not on campus for a duration of 8 to 9 months. This meant those students had greatly reduced clinical exposure and hence their continual learning might have been negatively affected.

The timing of the distribution of the survey should be equal in every batch. For example, the survey should either be administered at the beginning of every school year, or at the end to get a fair estimation of how cross-cultural competence improves over the years. However, there was a lack of unity in start and end dates across all 5 years due to the COVID-19 pandemic.

In our study, we asked students to assess their cross-cultural competence based on their individual perspectives. Therefore, an aspect of subjectivity exists while answering the questions. The results generated were dependent on how students perceive their knowledge and capabilities which varied greatly from student to student.

## Conclusion

Identification of such themes helped us recognize which issues medical students struggled with the most. With the help of the data collected, more emphasis can then be suggested on medical students' longitudinal training that will improve the quality of care being provided. This is a first of its kind survey done in Pakistan to assess cross-cultural competence of medical students at a private medical university in Karachi, Pakistan. As expected from previous studies, results in this study also showed that cross-cultural competence increases as medical students get exposed to more patients and receive further training over the years. An upward trend in skill, knowledge and comfort level shows that training being provided does, indeed, enable medical students to care for patients from different backgrounds. This study highlights areas of weakness that medical students may face when caring for patients coming from diverse populations. It provides a base for future studies aiming to explore cross-cultural competence in students. This study can also be used to assess the needs for training in similar settings such as other medical universities in Pakistan. 

## Supplementary Information

**Additional file 1.**

## Data Availability

All data generated or analysed during this study are included in this published article [and its supplementary information files].

## Abbreviations

SC: Sociocultural
AKUH: Aga Khan University Hospital
