# Supplementary material for: Assessing medical students’ perception of cross-cultural competence at a private University in Karachi
Source: BMC Med Educ. 2022 Jul 9;22:534. doi: 10.1186/s12909-022-03588-0 (PMC9270822; doi:10.1186/s12909-022-03588-0)
Supplement: Supplementary file 1 — Additional file 1. [file 12909_2022_3588_MOESM1_ESM.docx]

**SUPPLEMENTARY MATERIAL**

**Table 1.1**

| **Gender** | | | |
| --- | --- | --- | --- |
|  | **Frequency** | **Percent** | **Cumulative Percent** |
| **Female** | **114** | **42.5** | **42.5** |
| **Male** | **152** | **56.7** | **100.0** |
| **I prefer not to say** | **2** | **.7** | **43.3** |
| **Total** | **268** | **100.0** |  |

| **Table 1.2**    **Year of Study** | | | |
| --- | --- | --- | --- |
|  | **Frequency** | **Percent** | **Cumulative Percent** |
| **Year 1** | **51** | **19.0** | **19.0** |
| **Year 2** | **52** | **19.4** | **38.4** |
| **Year 3** | **47** | **17.5** | **56.0** |
| **Year 4** | **66** | **24.6** | **80.6** |
| **Year 5** | **52** | **19.4** | **100.0** |
| **Total** | **268** | **100.0** |  |

|  | **I: To recognize factors that predisposes to disease** | | | | | | | | |
| --- | --- | --- | --- | --- | --- | --- | --- | --- | --- |
|  | **Extremely** | | **Mildly** | | **Moderately** | | **Not at all** | | **Very** |
|  | **Mean** | **Standard Deviation** | **Mean** | **Standard Deviation** | **Mean** | **Standard Deviation** | **Mean** | **Standard Deviation** | **Standard Deviation** |
| **Knowledge: SC** | **3.58** | **.70** | **2.77** | **.48** | **3.23** | **.50** | **3.50** | **.14** | **.56** |
| **Comfort: SC** | **3.60** | **.73** | **2.46** | **.29** | **3.01** | **.63** | **2.88** | **.18** | **.75** |
| **Skill: SC** | **3.06** | **.95** | **2.67** | **.26** | **2.76** | **.54** | **2.75** | **.71** | **.81** |
| **Knowledge: Religion** | **3.65** | **.83** | **2.88** | **.52** | **3.12** | **.62** | **3.13** | **.18** | **.60** |
| **Comfort: Religion** | **3.66** | **.86** | **2.50** | **.61** | **3.04** | **.64** | **3.00** | **.00** | **.71** |
| **Skill: Religion** | **3.21** | **1.04** | **3.00** | **.35** | **2.92** | **.58** | **2.00** | **.35** | **.84** |
| **Knowledge: Alternative Medicine** | **3.16** | **.98** | **2.83** | **.41** | **3.28** | **.82** | **2.00** | **.71** | **.79** |
| **Comfort: Alternative Medicine** | **3.19** | **.88** | **2.67** | **.41** | **2.90** | **.63** | **2.00** | **.71** | **.83** |
| **Skill: Alternative Medicine** | **2.71** | **.96** | **2.50** | **.63** | **2.83** | **.62** | **2.25** | **.35** | **.89** |
| **Comfort: Language** | **2.86** | **.82** | **2.88** | **.38** | **2.83** | **.74** | **3.00** | **.35** | **.66** |
| **Skill: Language** | **2.53** | **.94** | **2.42** | **.41** | **2.64** | **.78** | **2.50** | **.35** | **.81** |

|  | **I: Considerate of patient's culture** | | | | | | | | | |
| --- | --- | --- | --- | --- | --- | --- | --- | --- | --- | --- |
|  | **Extremely** | | **Mildly** | | **Moderately** | | **Not at all** | | **Very** | |
|  | **Mean** | **Standard Deviation** | **Mean** | **Standard Deviation** | **Mean** | **Standard Deviation** | **Mean** | **Standard Deviation** | **Mean** | **Standard Deviation** |
| **Knowledge: SC** | **3.63** | **.68** | **3.09** | **.32** | **3.26** | **.59** | **3.50** | **.14** | **3.39** | **.62** |
| **Comfort: SC** | **3.70** | **.72** | **2.57** | **.66** | **3.09** | **.64** | **1.88** | **1.24** | **3.28** | **.67** |
| **Skill: SC** | **3.12** | **.96** | **2.57** | **.28** | **2.87** | **.51** | **3.50** | **.35** | **2.89** | **.84** |
| **Knowledge: Religion** | **3.66** | **.81** | **3.18** | **.57** | **3.18** | **.60** | **3.50** | **.71** | **3.53** | **.72** |
| **Comfort: Religion** | **3.77** | **.82** | **2.54** | **.57** | **3.11** | **.67** | **2.50** | **.71** | **3.36** | **.77** |
| **Skill: Religion** | **3.28** | **.99** | **2.61** | **.50** | **3.06** | **.44** | **2.00** | **.35** | **3.07** | **.99** |
| **Knowledge: Alternative Medicine** | **3.28** | **.99** | **3.07** | **.98** | **3.38** | **.72** | **1.75** | **1.06** | **3.04** | **.82** |
| **Comfort: Alternative Medicine** | **3.32** | **.88** | **2.29** | **.70** | **3.07** | **.65** | **1.75** | **1.06** | **2.93** | **.74** |
| **Skill: Alternative Medicine** | **2.80** | **.96** | **2.71** | **.70** | **2.97** | **.48** | **1.50** | **.71** | **2.55** | **.88** |
| **Comfort: Language** | **2.92** | **.84** | **2.64** | **.70** | **2.92** | **.64** | **2.88** | **.18** | **2.67** | **.67** |
| **Skill: Language** | **2.58** | **.96** | **2.14** | **.43** | **2.75** | **.65** | **2.63** | **.18** | **2.43** | **.85** |

|  | **A: more comfortable with patients from different background** | | | | | | | | | |
| --- | --- | --- | --- | --- | --- | --- | --- | --- | --- | --- |
|  | **Agree** | | **Disagree** | | **Neutral** | | **Strongly agree** | | **Strongly disagree** | |
|  | **Mean** | **Std. Dev.** | **Mean** | **Std. Dev.** | **Mean** | **Std. Dev.** | **Mean** | **Std. Dev.** | **Mean** | **Std. Dev.** |
| **Knowledge: SC** | **3.45** | **.56** | **3.37** | **.76** | **3.43** | **.67** | **3.66** | **.73** | **3.75** | **.66** |
| **Comfort: SC** | **3.35** | **.71** | **3.55** | **.91** | **3.39** | **.72** | **3.55** | **.81** | **3.88** | **.78** |
| **Skill: SC** | **2.98** | **.75** | **2.78** | **1.01** | **3.00** | **.89** | **3.12** | **.94** | **3.19** | **1.18** |
| **Knowledge: Religion** | **3.47** | **.74** | **3.23** | **.74** | **3.50** | **.73** | **3.86** | **.77** | **3.94** | **.94** |
| **Comfort: Religion** | **3.39** | **.80** | **3.52** | **1.05** | **3.50** | **.79** | **3.65** | **.80** | **4.19** | **.55** |
| **Skill: Religion** | **3.11** | **.91** | **2.97** | **.92** | **3.16** | **.89** | **3.32** | **1.04** | **3.06** | **1.39** |
| **Knowledge: Alternative Medicine** | **3.11** | **.93** | **2.95** | **1.06** | **3.26** | **.84** | **3.34** | **.88** | **3.63** | **.75** |
| **Comfort: Alternative Medicine** | **2.92** | **.80** | **3.34** | **1.06** | **3.18** | **.71** | **3.21** | **.88** | **3.88** | **.63** |
| **Skill: Alternative Medicine** | **2.59** | **.81** | **2.45** | **.99** | **2.94** | **.87** | **2.82** | **.95** | **2.50** | **1.00** |
| **Comfort: Language** | **2.67** | **.73** | **3.07** | **.55** | **2.94** | **.75** | **2.78** | **.87** | **3.25** | **.68** |
| **Skill: Language** | **2.46** | **.85** | **2.69** | **.92** | **2.60** | **.86** | **2.54** | **.91** | **2.50** | **1.29** |

|  | **A: aware of own biases and limitations** | | | | | | | | | |
| --- | --- | --- | --- | --- | --- | --- | --- | --- | --- | --- |
|  | **Agree** | | **Disagree** | | **Neutral** | | **Strongly agree** | | **Strongly disagree** | |
|  | **Mean** | **Std.**  **Dev.** | **Mean** | **Std.**  **Dev.** | **Mean** | **Std.**  **Dev.** | **Mean** | **Std.**  **Dev.** | **Mean** | **Std.**  **Dev.** |
| **Knowledge: SC** | **3.59** | **.61** | **3.13** | **.73** | **3.23** | **.62** | **3.67** | **.68** | **3.00** | **.57** |
| **Comfort: SC** | **3.53** | **.73** | **2.98** | **.77** | **3.24** | **.70** | **3.57** | **.85** | **2.88** | **.18** |
| **Skill: SC** | **3.11** | **.84** | **2.58** | **1.09** | **2.81** | **.79** | **3.09** | **.94** | **2.25** | **.00** |
| **Knowledge: Religion** | **3.63** | **.71** | **3.19** | **.82** | **3.25** | **.76** | **3.84** | **.79** | **3.00** | **.35** |
| **Comfort: Religion** | **3.63** | **.79** | **3.06** | **1.10** | **3.24** | **.76** | **3.64** | **.89** | **2.75** | **.35** |
| **Skill: Religion** | **3.23** | **.91** | **2.77** | **1.14** | **2.99** | **.84** | **3.29** | **1.09** | **2.38** | **.18** |
| **Knowledge:**  **Alternative Medicine** | **3.26** | **.88** | **2.42** | **.90** | **3.09** | **.89** | **3.40** | **.91** | **2.00** | **.71** |
| **Comfort:**  **Alternative Medicine** | **3.17** | **.79** | **2.50** | **.80** | **3.00** | **.82** | **3.33** | **.91** | **2.00** | **.71** |
| **Skill:**  **Alternative Medicine** | **2.78** | **.81** | **1.79** | **.78** | **2.63** | **.88** | **2.93** | **1.06** | **2.75** | **.35** |
| **Comfort: Language** | **2.81** | **.71** | **2.29** | **.83** | **2.78** | **.71** | **3.11** | **.89** | **2.88** | **.53** |
| **Skill: Language** | **2.57** | **.82** | **1.98** | **.97** | **2.47** | **.90** | **2.73** | **.96** | **2.13** | **.18** |

|  | **A: Sociocultural backgrounds are important to incorporate into care** | | | | | | | |
| --- | --- | --- | --- | --- | --- | --- | --- | --- |
|  | **Agree** | | **Disagree** | | **Neutral** | | **Strongly agree** | |
|  | **Mean** | **Std. Dev.** | **Mean** | **Std. Dev.** | **Mean** | **Std. Dev.** | **Mean** | **Std. Dev.** |
| **Knowledge: SC** | **3.50** | **.60** | **3.31** | **.54** | **3.37** | **.60** | **3.52** | **.74** |
| **Comfort: SC** | **3.39** | **.76** | **2.79** | **.86** | **3.21** | **.71** | **3.61** | **.72** |
| **Skill: SC** | **2.96** | **.86** | **2.89** | **.70** | **3.01** | **.73** | **3.05** | **.93** |
| **Knowledge: Religion** | **3.51** | **.78** | **3.21** | **.67** | **3.37** | **.64** | **3.66** | **.78** |
| **Comfort: Religion** | **3.49** | **.87** | **3.14** | **.67** | **3.19** | **.70** | **3.65** | **.80** |
| **Skill: Religion** | **3.16** | **.94** | **3.00** | **.69** | **3.13** | **.68** | **3.16** | **1.03** |
| **Knowledge: Alternative Medicine** | **3.07** | **.88** | **3.43** | **.73** | **3.56** | **.72** | **3.18** | **.99** |
| **Comfort: Alternative Medicine** | **3.06** | **.81** | **2.86** | **.85** | **3.22** | **.68** | **3.16** | **.91** |
| **Skill: Alternative Medicine** | **2.67** | **.85** | **3.14** | **.75** | **2.97** | **.72** | **2.66** | **1.00** |
| **Comfort: Language** | **2.77** | **.70** | **3.18** | **.75** | **2.99** | **.82** | **2.81** | **.80** |
| **Skill: Language** | **2.45** | **.82** | **3.07** | **.84** | **2.92** | **.87** | **2.47** | **.91** |

|  | **A: understand the need to avoid stereotyping** | | | | |
| --- | --- | --- | --- | --- | --- |
|  | **Agree** | **Disagree** | **Neutral** | **Strongly agree** | **Strongly disagree** |
|  | **Mean** | **Mean** | **Mean** | **Mean** | **Mean** |
| **Knowledge: SC** | **3.54** | **2.80** | **3.34** | **3.51** | **2.60** |
| **Comfort: SC** | **3.37** | **2.17** | **3.04** | **3.62** | **2.75** |
| **Skill: SC** | **2.97** | **2.42** | **3.01** | **3.03** | **2.25** |
| **Knowledge: Religion** | **3.62** | **2.67** | **3.23** | **3.59** | **2.75** |
| **Comfort: Religion** | **3.52** | **2.50** | **3.06** | **3.64** | **2.50** |
| **Skill: Religion** | **3.25** | **2.33** | **3.09** | **3.12** | **2.50** |
| **Knowledge: Alternative Medicine** | **3.21** | **2.83** | **3.28** | **3.16** | **2.50** |
| **Comfort: Alternative Medicine** | **3.11** | **2.00** | **2.95** | **3.19** | **2.50** |
| **Skill: Alternative Medicine** | **2.75** | **2.83** | **2.87** | **2.65** | **3.00** |
| **Comfort: Language** | **2.76** | **2.50** | **2.99** | **2.82** | **2.50** |
| **Skill: Language** | **2.59** | **2.17** | **2.76** | **2.46** | **2.00** |

|  | **A: Appreciate the diversity within cultural groups** | | | | |
| --- | --- | --- | --- | --- | --- |
|  | **Agree** | **Disagree** | **Neutral** | **Strongly agree** | **Strongly disagree** |
|  | **Mean** | **Mean** | **Mean** | **Mean** | **Mean** |
| **Knowledge: SC** | **3.49** | **3.30** | **3.34** | **3.53** | **2.60** |
| **Comfort: SC** | **3.38** | **2.54** | **3.07** | **3.62** | **2.75** |
| **Skill: SC** | **2.99** | **2.67** | **3.00** | **3.03** | **2.25** |
| **Knowledge: Religion** | **3.56** | **2.42** | **3.39** | **3.62** | **2.75** |
| **Comfort: Religion** | **3.49** | **2.54** | **3.11** | **3.66** | **2.50** |
| **Skill: Religion** | **3.20** | **2.50** | **3.05** | **3.17** | **2.50** |
| **Knowledge: Alternative Medicine** | **3.13** | **3.00** | **3.28** | **3.23** | **2.50** |
| **Comfort: Alternative Medicine** | **3.06** | **2.42** | **2.92** | **3.25** | **2.50** |
| **Skill: Alternative Medicine** | **2.71** | **2.33** | **2.92** | **2.69** | **3.00** |
| **Comfort: Language** | **2.77** | **2.67** | **2.98** | **2.85** | **2.50** |
| **Skill: Language** | **2.57** | **2.58** | **2.84** | **2.44** | **2.00** |

| **Group Statistics** | | | | | |
| --- | --- | --- | --- | --- | --- |
|  | **Year of Study** | **N** | **Mean** | **Std. Deviation** | **Std. Error Mean** |
| **Knowledge: SC** | **Year 1** | **51** | **14.6275** | **3.59422** | **.50329** |
|  | **Year 5** | **52** | **19.0769** | **2.84809** | **.39496** |
| **Comfort: SC** | **Year 1** | **51** | **13.1765** | **2.92374** | **.40941** |
|  | **Year 5** | **52** | **14.6154** | **2.99774** | **.41571** |
| **Skills: SC** | **Year 1** | **51** | **9.1373** | **3.77899** | **.52916** |
|  | **Year 5** | **52** | **14.1346** | **2.93744** | **.40735** |
| **Knowledge: R** | **Year 1** | **51** | **11.9608** | **3.11102** | **.43563** |
|  | **Year 5** | **52** | **15.6538** | **2.23944** | **.31055** |
| **Comfort: R** | **Year 1** | **51** | **13.3922** | **3.86305** | **.54093** |
|  | **Year 5** | **52** | **15.0000** | **3.19313** | **.44281** |
| **Skills: R** | **Year 1** | **51** | **9.1569** | **3.58537** | **.50205** |
|  | **Year 5** | **52** | **14.8654** | **3.03592** | **.42101** |
| **Knowledge: AM** | **Year 1** | **51** | **5.4118** | **2.08975** | **.29262** |
|  | **Year 5** | **52** | **7.0577** | **1.58935** | **.22040** |
| **Comfort: AM** | **Year 1** | **51** | **6.3725** | **1.84348** | **.25814** |
|  | **Year 5** | **52** | **6.5962** | **1.43157** | **.19852** |
| **Skills: AM** | **Year 1** | **51** | **4.1961** | **1.99017** | **.27868** |
|  | **Year 5** | **52** | **6.3269** | **1.43788** | **.19940** |
| **Comfort: L** | **Year 1** | **51** | **10.4510** | **3.06799** | **.42960** |
|  | **Year 5** | **52** | **11.8654** | **3.73656** | **.51817** |
| **Skills: L** | **Year 1** | **51** | **7.7451** | **3.77276** | **.52829** |
|  | **Year 5** | **52** | **11.6154** | **3.74246** | **.51899** |

|  | | **Levene's Test for Equality of Variances** | | **t-test for Equality of Means** | | | | |
| --- | --- | --- | --- | --- | --- | --- | --- | --- |
|  |  | **F** | **Sig.** | **t** | **df** | **Sig. (2-tailed)** | **Mean Difference** | **Std. Error Difference** |
|  |  |  |  |  |  |  |  |  |
| **knowledge: SC** | **Equal variances assumed** | **1.362** | **.246** | **-6.970** | **101** | **.000** | **-4.44947** | **.63833** |
|  | **Equal variances not assumed** |  |  | **-6.955** | **95.163** | **.000** | **-4.44947** | **.63976** |
| **Comfort: SC** | **Equal variances assumed** | **.112** | **.738** | **-2.466** | **101** | **.015** | **-1.43891** | **.58361** |
|  | **Equal variances not assumed** |  |  | **-2.466** | **100.997** | **.015** | **-1.43891** | **.58346** |
| **Skills: SC** | **Equal variances assumed** | **3.156** | **.079** | **-7.502** | **101** | **.000** | **-4.99736** | **.66618** |
|  | **Equal variances not assumed** |  |  | **-7.483** | **94.339** | **.000** | **-4.99736** | **.66779** |
| **Knowledge: R** | **Equal variances assumed** | **5.307** | **.023** | **-6.925** | **101** | **.000** | **-3.69306** | **.53333** |
|  | **Equal variances not assumed** |  |  | **-6.903** | **90.754** | **.000** | **-3.69306** | **.53499** |
| **Comfort: R** | **Equal variances assumed** | **3.865** | **.052** | **-2.304** | **101** | **.023** | **-1.60784** | **.69777** |
|  | **Equal variances not assumed** |  |  | **-2.300** | **96.833** | **.024** | **-1.60784** | **.69906** |
| **Skills: R** | **Equal variances assumed** | **1.988** | **.162** | **-8.727** | **101** | **.000** | **-5.70852** | **.65415** |
|  | **Equal variances not assumed** |  |  | **-8.712** | **97.687** | **.000** | **-5.70852** | **.65521** |
| **Knowledge: AM** | **Equal variances assumed** | **4.855** | **.030** | **-4.505** | **101** | **.000** | **-1.64593** | **.36538** |
|  | **Equal variances not assumed** |  |  | **-4.493** | **93.364** | **.000** | **-1.64593** | **.36634** |
| **Comfort: AM** | **Equal variances assumed** | **4.457** | **.037** | **-.688** | **101** | **.493** | **-.22360** | **.32486** |
|  | **Equal variances not assumed** |  |  | **-.687** | **94.296** | **.494** | **-.22360** | **.32565** |
| **Skills: AM** | **Equal variances assumed** | **2.572** | **.112** | **-6.238** | **101** | **.000** | **-2.13084** | **.34162** |
|  | **Equal variances not assumed** |  |  | **-6.218** | **90.934** | **.000** | **-2.13084** | **.34267** |
| **Comfort: L** | **Equal variances assumed** | **2.647** | **.107** | **-2.097** | **101** | **.038** | **-1.41440** | **.67438** |
|  | **Equal variances not assumed** |  |  | **-2.101** | **97.986** | **.038** | **-1.41440** | **.67310** |
| **Skills: L** | **Equal variances assumed** | **.218** | **.642** | **-5.227** | **101** | **.000** | **-3.87029** | **.74051** |
|  | **Equal variances not assumed** |  |  | **-5.226** | **100.923** | **.000** | **-3.87029** | **.74057** |

| **Group Statistics** | | | | | |
| --- | --- | --- | --- | --- | --- |
|  | **Language** | **N** | **Mean** | **Std. Deviation** | **Std. Error Mean** |
| **knowledge: SC** | **Urdu** | **150** | **17.3267** | **3.13777** | **.25620** |
|  | **Other th** | **118** | **17.5339** | **3.42859** | **.31563** |
| **Comfort: SC** | **Urdu** | **150** | **13.6000** | **2.70719** | **.22104** |
|  | **Other th** | **118** | **13.8814** | **3.40480** | **.31344** |
| **Skills: SC** | **Urdu** | **150** | **11.8400** | **3.33394** | **.27222** |
|  | **Other th** | **118** | **12.1780** | **3.60823** | **.33216** |
| **Knowledge: R** | **Urdu** | **150** | **13.9400** | **3.04823** | **.24889** |
|  | **Other th** | **118** | **14.4237** | **3.04498** | **.28031** |
| **Comfort: R** | **Urdu** | **150** | **13.8867** | **3.19508** | **.26088** |
|  | **Other th** | **118** | **14.1186** | **3.47437** | **.31984** |
| **Skills: R** | **Urdu** | **150** | **12.4200** | **3.66604** | **.29933** |
|  | **Other th** | **118** | **12.8305** | **3.83708** | **.35323** |
| **Knowledge: AM** | **Urdu** | **150** | **6.3800** | **1.87770** | **.15331** |
|  | **Other th** | **118** | **6.3729** | **1.75799** | **.16184** |
| **Comfort: AM** | **Urdu** | **150** | **6.1200** | **1.66254** | **.13575** |
|  | **Other th** | **118** | **6.3559** | **1.67702** | **.15438** |
| **Skills: AM** | **Urdu** | **150** | **5.3933** | **1.73745** | **.14186** |
|  | **Other th** | **118** | **5.5000** | **1.84784** | **.17011** |
| **Comfort: L** | **Urdu** | **150** | **11.4667** | **2.97792** | **.24315** |
|  | **Other th** | **118** | **11.0593** | **3.08925** | **.28439** |
| **Skills: L** | **Urdu** | **150** | **10.1600** | **3.47779** | **.28396** |
|  | **Other th** | **118** | **10.1695** | **3.54773** | **.32660** |

| **Independent Samples Test** | | | | | | | | |
| --- | --- | --- | --- | --- | --- | --- | --- | --- |
|  | | **Levene's Test for Equality of Variances** | | **t-test for Equality of Means** | | | | |
|  |  | **F** | **Sig.** | **t** | **df** | **Sig. (2-tailed)** | **Mean Difference** | **Std. Error Difference** |
|  |  |  |  |  |  |  |  |  |
| **knowledge: SC** | **Equal variances assumed** | **1.267** | **.261** | **-.515** | **266** | **.607** | **-.20723** | **.40223** |
|  | **Equal variances not assumed** |  |  | **-.510** | **240.116** | **.611** | **-.20723** | **.40652** |
| **Comfort: SC** | **Equal variances assumed** | **4.787** | **.030** | **-.754** | **266** | **.452** | **-.28136** | **.37332** |
|  | **Equal variances not assumed** |  |  | **-.734** | **219.653** | **.464** | **-.28136** | **.38354** |
| **Skills: SC** | **Equal variances assumed** | **.001** | **.978** | **-.794** | **266** | **.428** | **-.33797** | **.42542** |
|  | **Equal variances not assumed** |  |  | **-.787** | **241.422** | **.432** | **-.33797** | **.42946** |
| **Knowledge: R** | **Equal variances assumed** | **.364** | **.547** | **-1.290** | **266** | **.198** | **-.48373** | **.37491** |
|  | **Equal variances not assumed** |  |  | **-1.290** | **251.467** | **.198** | **-.48373** | **.37486** |
| **Comfort: R** | **Equal variances assumed** | **.983** | **.322** | **-.568** | **266** | **.571** | **-.23198** | **.40863** |
|  | **Equal variances not assumed** |  |  | **-.562** | **240.778** | **.575** | **-.23198** | **.41274** |
| **Skills: R** | **Equal variances assumed** | **.295** | **.587** | **-.891** | **266** | **.373** | **-.41051** | **.46048** |
|  | **Equal variances not assumed** |  |  | **-.887** | **245.828** | **.376** | **-.41051** | **.46300** |
| **Knowledge: AM** | **Equal variances assumed** | **1.047** | **.307** | **.032** | **266** | **.975** | **.00712** | **.22469** |
|  | **Equal variances not assumed** |  |  | **.032** | **258.041** | **.975** | **.00712** | **.22293** |
| **Comfort: AM** | **Equal variances assumed** | **.078** | **.780** | **-1.149** | **266** | **.252** | **-.23593** | **.20536** |
|  | **Equal variances not assumed** |  |  | **-1.148** | **250.346** | **.252** | **-.23593** | **.20557** |
| **Skills: AM** | **Equal variances assumed** | **.168** | **.682** | **-.485** | **266** | **.628** | **-.10667** | **.21987** |
|  | **Equal variances not assumed** |  |  | **-.482** | **243.754** | **.631** | **-.10667** | **.22150** |
| **Comfort: L** | **Equal variances assumed** | **.190** | **.663** | **1.093** | **266** | **.275** | **.40734** | **.37252** |
|  | **Equal variances not assumed** |  |  | **1.089** | **246.952** | **.277** | **.40734** | **.37416** |
| **Skills: L** | **Equal variances assumed** | **.263** | **.608** | **-.022** | **266** | **.982** | **-.00949** | **.43175** |
|  | **Equal variances not assumed** |  |  | **-.022** | **249.014** | **.983** | **-.00949** | **.43278** |

| **Group Statistics** | | | | | |
| --- | --- | --- | --- | --- | --- |
|  | **Origin** | **N** | **Mean** | **Std. Deviation** | **Std. Error Mean** |
| **knowledge: SC** | **Pakistan** | **246** | **17.4024** | **3.21042** | **.20469** |
|  | **Other th** | **22** | **17.5909** | **3.89944** | **.83136** |
| **Comfort: SC** | **Pakistan** | **246** | **13.6870** | **3.03375** | **.19343** |
|  | **Other th** | **22** | **14.1364** | **3.04405** | **.64899** |
| **Skills: SC** | **Pakistan** | **246** | **11.8821** | **3.48968** | **.22249** |
|  | **Other th** | **22** | **13.1818** | **2.83912** | **.60530** |
| **Knowledge: R** | **Pakistan** | **246** | **14.2073** | **3.08414** | **.19664** |
|  | **Other th** | **22** | **13.5455** | **2.63181** | **.56110** |
| **Comfort: R** | **Pakistan** | **246** | **14.0447** | **3.37004** | **.21487** |
|  | **Other th** | **22** | **13.3636** | **2.62851** | **.56040** |
| **Skills: R** | **Pakistan** | **246** | **12.5163** | **3.81802** | **.24343** |
|  | **Other th** | **22** | **13.5455** | **2.61365** | **.55723** |
| **Knowledge: AM** | **Pakistan** | **246** | **6.3293** | **1.85641** | **.11836** |
|  | **Other th** | **22** | **6.9091** | **1.30600** | **.27844** |
| **Comfort: AM** | **Pakistan** | **246** | **6.2033** | **1.67068** | **.10652** |
|  | **Other th** | **22** | **6.4545** | **1.68261** | **.35873** |
| **Skills: AM** | **Pakistan** | **246** | **5.4309** | **1.81415** | **.11567** |
|  | **Other th** | **22** | **5.5455** | **1.43849** | **.30669** |
| **Comfort: L** | **Pakistan** | **246** | **11.2846** | **3.02917** | **.19313** |
|  | **Other th** | **22** | **11.3182** | **3.09202** | **.65922** |
| **Skills: L** | **Pakistan** | **246** | **10.0447** | **3.48203** | **.22201** |
|  | **Other th** | **22** | **11.5000** | **3.52879** | **.75234** |

| **Independent Samples Test** | | | | | | | | | |  |
| --- | --- | --- | --- | --- | --- | --- | --- | --- | --- | --- |
|  | | **Levene's Test for Equality of Variances** | | **t-test for Equality of Means** | | | | | |  |
|  |  | **F** | **Sig.** | **t** | **df** | **Sig. (2-tailed)** | **Mean Difference** | **Std. Error Difference** |  |  |
|  |  |  |  |  |  |  |  |  |  |  |
| **knowledge: SC** | **Equal variances assumed** | **1.345** | **.247** | **-.259** | **266** | **.796** | **-.18847** | **.72769** |  |  |
|  | **Equal variances not assumed** |  |  | **-.220** | **23.616** | **.828** | **-.18847** | **.85619** |  |  |
| **Comfort: SC** | **Equal variances assumed** | **.072** | **.789** | **-.665** | **266** | **.506** | **-.44937** | **.67528** |  |  |
|  | **Equal variances not assumed** |  |  | **-.664** | **24.880** | **.513** | **-.44937** | **.67720** |  |  |
| **Skills: SC** | **Equal variances assumed** | **.745** | **.389** | **-1.696** | **266** | **.091** | **-1.29970** | **.76612** |  |  |
|  | **Equal variances not assumed** |  |  | **-2.015** | **27.016** | **.054** | **-1.29970** | **.64490** |  |  |
| **Knowledge: R** | **Equal variances assumed** | **.554** | **.457** | **.975** | **266** | **.331** | **.66186** | **.67891** |  |  |
|  | **Equal variances not assumed** |  |  | **1.113** | **26.441** | **.276** | **.66186** | **.59456** |  |  |
| **Comfort: R** | **Equal variances assumed** | **1.549** | **.214** | **.923** | **266** | **.357** | **.68108** | **.73825** |  |  |
|  | **Equal variances not assumed** |  |  | **1.135** | **27.577** | **.266** | **.68108** | **.60018** |  |  |
| **Skills: R** | **Equal variances assumed** | **3.116** | **.079** | **-1.238** | **266** | **.217** | **-1.02919** | **.83161** |  |  |
|  | **Equal variances not assumed** |  |  | **-1.693** | **29.687** | **.101** | **-1.02919** | **.60808** |  |  |
| **Knowledge: AM** | **Equal variances assumed** | **2.990** | **.085** | **-1.432** | **266** | **.153** | **-.57982** | **.40479** |  |  |
|  | **Equal variances not assumed** |  |  | **-1.916** | **29.193** | **.065** | **-.57982** | **.30255** |  |  |
| **Comfort: AM** | **Equal variances assumed** | **.278** | **.599** | **-.676** | **266** | **.500** | **-.25129** | **.37199** |  |  |
|  | **Equal variances not assumed** |  |  | **-.672** | **24.850** | **.508** | **-.25129** | **.37421** |  |  |
| **Skills: AM** | **Equal variances assumed** | **2.757** | **.098** | **-.288** | **266** | **.774** | **-.11456** | **.39774** |  |  |
|  | **Equal variances not assumed** |  |  | **-.350** | **27.351** | **.729** | **-.11456** | **.32777** |  |  |
| **Comfort: L** | **Equal variances assumed** | **.162** | **.687** | **-.050** | **266** | **.960** | **-.03363** | **.67519** |  |  |
|  | **Equal variances not assumed** |  |  | **-.049** | **24.744** | **.961** | **-.03363** | **.68693** |  |  |
| **Skills: L** | **Equal variances assumed** | **.008** | **.927** | **-1.876** | **266** | **.062** | **-1.45528** | **.77568** |  |  |
|  | **Equal variances not assumed** |  |  | **-1.855** | **24.800** | **.075** | **-1.45528** | **.78441** |  |  |
